# Cre-Activation in ErbB4-Positive Neurons of Floxed *Grin1*/NMDA Receptor Mice Is Not Associated With Major Behavioral Impairment

**DOI:** 10.3389/fpsyt.2021.750106

**Published:** 2021-11-25

**Authors:** Anne S. Mallien, Natascha Pfeiffer, Miriam A. Vogt, Sabine Chourbaji, Rolf Sprengel, Peter Gass, Dragos Inta

**Affiliations:** ^1^Department of Psychiatry and Psychotherapy, Medical Faculty Mannheim, RG Animal Models in Psychiatry, Central Institute of Mental Health, Heidelberg University, Mannheim, Germany; ^2^Interfaculty Biomedical Research Facility (IBF), Heidelberg University, Heidelberg, Germany; ^3^Research Group of the Max Planck Institute for Medical Research at the Institute for Anatomy and Cell Biology, Heidelberg University, Heidelberg, Germany; ^4^Interdisciplinary Center for Neurosciences (IZN), Heidelberg University, Heidelberg, Germany; ^5^Department of Psychiatry (UPK), University of Basel, Basel, Switzerland

**Keywords:** glutamate, neurodevelopment, pharmacogenetic, neuregulin-1, schizophrenia, NMDA receptor, post-adolescent

## Abstract

Extensive evidence suggests a dysfunction of the glutamate NMDA receptor (NMDAR) in schizophrenia, a severe psychiatric disorder with putative early neurodevelopmental origins, but clinical onset mainly during late adolescence. On the other hand, pharmacological models using NMDAR antagonists and the clinical manifestation of anti-NMDAR encephalitis indicate that NMDAR blockade/hypofunction can trigger psychosis also at adult stages, without any early developmental dysfunction. Previous genetic models of NMDAR hypofunction restricted to parvalbumin-positive interneurons indicate the necessity of an early postnatal impairment to trigger schizophrenia-like abnormalities, whereas the cellular substrates of NMDAR-mediated psychosis at adolescent/adult stages are unknown. Neuregulin 1 (NRG1) and its receptor ErbB4 represent schizophrenia-associated susceptibility factors that closely interact with NMDAR. To determine the neuronal populations implicated in “late” NMDAR-driven psychosis, we analyzed the effect of the inducible ablation of NMDARs in *ErbB4*-expressing cells in mice during late adolescence using a pharmacogenetic approach. Interestingly, the tamoxifen-inducible NMDAR deletion during this late developmental stage did not induce behavioral alterations resembling depression, schizophrenia or anxiety. Our data indicate that post-adolescent NMDAR deletion, even in a wider cell population than parvalbumin-positive interneurons, is also not sufficient to generate behavioral abnormalities resembling psychiatric disorders. Other neuronal substrates that have to be revealed by future studies, may underlie post-adolescent NMDAR-driven psychosis.

## Introduction

Despite intense research, the molecular and cellular mechanisms of psychotic disorders, like schizophrenia and anti-NMDA receptor (NMDAR) encephalitis that emerge often during post-adolescence/young adulthood, are only partly understood. Glutamate is the main excitatory neurotransmitter in the mammalian brain. NMDAR represent one of the ligand-gated non-selective ionotropic glutamate receptors, which are widely present throughout the brain, in high density within the hippocampus and the cerebral cortex (1). NMDAR are preferentially expressed in excitatory neurons that represent about 70% of the neurons containing NMDAR (1). Nevertheless, GABAergic interneurons express as well NMDAR, numerous onto parvalbumin (PV)-positive interneurons that show a particularly strong glutamatergic input (2). Extensive evidence implicates dysfunction of the glutamate NMDAR in the emergence of psychotic symptoms (3). The glutamate hypothesis of schizophrenia is the most influential alternative explanatory model of schizophrenia, postulating hypofunction of NMDAR as pathophysiological mechanism (4). It emerged from observations that NMDAR antagonists (phencyclidine/PCP, ketamine, MK-801) mimic better than any other psychotomimetic drug the whole spectrum of psychotic symptoms, i.e., not only positive, but also negative symptoms and cognitive deficits (5). Several studies reported NMDAR abnormalities in schizophrenia, showing reduced NMDAR expression in post-mortem brain tissue in schizophrenia (6, 7), diminished expression of NMDAR/associated proteins in induced pluripotent stem cell-derived (iPSC) neurons in schizophrenia (8), and increased cerebrospinal fluid and post-stress levels of kynurenic acid, an endogenous NMDAR antagonist in schizophrenia (9). In addition, proteins structurally and functionally closely linked to NMDAR, like NRG1 display strong positive genetic association with schizophrenia (10), whereas abnormal cortical oscillations triggered by NMDAR dysfunction (11) represent an electrophysiological endophenotype of schizophrenia (12). Moreover, subjects suffering from anti-NMDAR encephalitis show an initial psychotic phase often indistinguishable from schizophrenia; therefore, an estimated 77% of cases with anti-NMDAR encephalitis is initially misdiagnosed as schizophrenia (13). Patients with anti-NMDAR encephalitis produce anti-GluN1 autoantibodies that reduce surface NMDAR clusters and protein in a titer-dependent fashion in rodents and humans *in vitro* and *in vivo* (14). Interestingly, the clinical manifestation of anti-NMDAR encephalitis shows age-dependent variations: autistic-like features during childhood (15), psychosis during young adulthood and less severe symptoms with predominant cognitive deficits in older patients (16). Moreover, the susceptibility to the psychotomimetic effects of NMDAR antagonists is minimal or absent in children and becomes maximal in early adulthood (17). In fact, the NMDR hypofunction hypothesis of schizophrenia is relying on initial clinical observations in adults. Although some rodent studies report protracted schizophrenia-like abnormalities following perinatal treatment with NMDAR antagonists (18), it appears clear that NMDAR hypofunction at young adult stages, without any previous developmental impairment, can induce as well abnormalities resembling psychosis. Animal models represent a useful experimental tool to clarify the role of abnormal NMDAR in psychosis-like abnormalities. Mice with reduced NMDAR expression (GluN1/*Grin1* knockdown, KD) that express 5–10% of the normal NMDAR levels, are viable and display schizophrenia-like abnormalities (19). However, this global NMDAR KD model does not allow the identification of the neuronal populations implicated in psychosis. Meanwhile conditional genetic models provide insights into these cell-specific mechanisms. Numerous data suggest that GABAergic interneurons play a central role in schizophrenia showing abnormal distribution and loss of subpopulations of GABAergic interneurons (20). Most studies focus on NMDAR hypofunction in fast-spiking PV-positive GABAergic interneurons that play a key role in generating cortical oscillatory activity (21). Abnormal synchronization of gamma-band activity may underlie cognitive deficits in schizophrenia (22).

However, mice with conditional ablation of NMDAR in PV-positive interneurons show largely normal behaviors (no hyperlocomotion and sensorimotor gating deficits as correlates of positive symptoms of schizophrenia), except for selective cognitive impairments (23, 24). Cre-driven recombination in these mice was detected in the somatosensory cortex and hippocampus at postnatal day 13 (P13) with about 80% recombination at 29 days (P29) (24). On contrary, mice with conditional ablation of NMDAR under the control of the Ppp1r2 (protein phosphatase 1, regulatory subunit 2) gene promoter, targeting mostly (about 75%), but not exclusively PV-positive interneurons, displayed schizophrenia-like abnormalities (25). Interestingly, these abnormalities were observed only in the mouse line in which Cre-driven recombination started at early postnatal stages, with NMDR expression absent in 40–50% of cortical and hippocampal interneurons in P28 mutant mice, but not in mice were recombination started at young adult stages (P56) (25). Therefore, NMDAR deficiency in PV-positive interneurons appears not sufficient to induce all psychosis-like features and if yes, only when occurring already at early postnatal stages and most likely, also in other neurons. However, the cellular substrates of NMDAR-driven psychosis at post-adolescent/young adult stages remain unknown, these results suggesting that a different neuronal population, larger than PV-positive interneurons may be implicated. In sum, there is a discrepancy between the currently available genetic models of NMDAR dysfunction, showing psychosis-like changes only when deleted at early postnatal stages, and pharmacological/clinical data, indicating that NMDAR blockade also/rather at later/even adult stages can induce psychosis. We hypothesized that if NMDAR deletion in PV-positive is insufficient to trigger psychosis-like changes during adulthood, extension to the larger population of ErbB4-positive cells may lead to such phenotype.

The schizophrenia-associated susceptibility factors that interact closely with NMDARs like neuregulin 1 (NRG1) and its receptor ErbB4, both main genetic risk factors associated with schizophrenia (26). Altered NRG1/ErbB4 signaling has been shown to contribute to NMDAR hypofunction in patients with schizophrenia (27) and mice with NRG1 deletion have 16% fewer functional NMDAR than wild-type mice, whereas if a similar change occurs also in ErbB4 KO mice was not determined (26). The expression pattern of ErbB4 is highly conserved during evolution from rodents to humans (28). ErbB4 mRNA is widely expressed throughout the adult brain, however, it is restricted in cortical regions to PV-positive interneurons (28). Considerable expression occurs in the subventricular zone (SVZ) and along the rostral migratory stream, as well as in other interneuronal clusters generated in the SVZ and potentially implicated in the pathophysiology of schizophrenia, forming the Islands of Calleja (ICj) (29, 30). Moreover, in the midbrain, ErbB4 mRNA expression is prominent in dopaminergic neurons in the substantia nigra pars compacta and adjacent ventral tegmental area (29). Further forebrain areas with ErbB4 expression are the septum, bed nucleus of stria terminalis, medial preoptic nucleus, suprachiasmatic nucleus, nucleus of the lateral olfactory tract, subthalamic nucleus, zona incerta, hypothalamus, pre- and supramammillary nuclei, the central gray, anterior pretectal nucleus and superior colliculus (29). In contrast, expression is minimal or absent in most areas of the thalamus, excepting the reticular nucleus and habenula (29).

We sought in the present study to delineate the specific contribution of NMDA receptors located on ErbB4-expressing neurons in the post-adolescent brain to abnormalities relevant for neuropsychiatric disorders by avoiding deleterious effects on early cortical circuitry by ablation of the obligatory GluN1 (formerly NR1) subunit of the NMDAR. The aim of our study is to identify the cellular substrates of psychosis induced by NMDAR hypofunction at post-adolescent stages, and not of schizophrenia in general (as a disease with most likely early neurodevelopmental impairment). We do not aim to find the cause of schizophrenia, but to determine if restricted ablation of NMDAR in a relevant cell population is associated with psychosis-like changes.

We employed the Cre/loxP recombination system and tamoxifen-controlled gene manipulation (31) for time- and cell type specific depletion of NMDARs during late adolescence in ErbB4-expressing neurons. Due to fast genetic inactivation of the functional *Grin1* mRNA expression within two weeks, the NMDAR signaling can be affected specifically in mature mice, avoiding any interference with earlier developmental brain circuitry formation. For the Tamoxifen-induced genetic NMDAR ablation we selected in mice the “late” developmental stage that corresponds to transition from adolescence to adulthood, which is the most frequent time of onset of both schizophrenia and anti-NMDAR encephalitis.

## Materials and Methods

### Mouse Lines Used in This Study

Mouse lines used in this study are available from the mouse repositories of the Jackson laboratories or the EMMA infrafrontier (*B6.129-Grin1*^*tm*2*Rsp*^/kctt, EM:09220; B6. *Cg*^*Erbb*4*tm*1.1(*cre*/*ERT*2)*Aibs*/*J*^, Stock: 012360; B6.Cg-*Gt(ROSA)26Sor*^*tm*14(*CAG*−*tdTomato*)*Hze*^ Stock 007914).

### Generation of *Grin1*^Δ*Erb*^ Mice and Induction of Cre-Mediated Recombination

To achieve NMDAR ablation specifically in most interneurons, we crossed the well-established *Grin1*^*f*/*f*^ line (32–34) with the tamoxifen inducible *ErbB4-CreERT2*-driver line (35).^.^ Mice harboring one copy of the *ErbB4-CreERT2 gene* and two copies of the *Grin1*^2*lox*^ (*Grin1*^*f*/*f*^) allele were used as cell-specific knockouts (herein called *Grin1*^*f*/*f*^*/ ErbB4-CreERT2*). Littermates without the *ErbB4-CreERT2* gene and only haploid or diploid the floxed *Grin1* allele (*Grin1*^*f*/+^ or *Grin1*^*f*/*lf*^) were used as controls (called hereafter *Grin1*^2*lox*^ or controls). We proved, the tamoxifen-induced interneuronal Cre activity by using tdTomato Cre reporter mice B6.Cg-*Gt(ROSA)26Sor*^*tm*14(*CAG*−*tdTomato*)*Hze*^, also known A14 (35). Mice were genotyped according to the public available resources of the mouse repositories: *A14*: (https://www.jax.org/Protocol?stockNumber=007914&protocolID=29436), *ErbB4-CreERT2*: (https://www.jax.org/Protocol?stockNumber=012360&protocolID=28814), and *Grin1*: for Grin1 genotyping the forward primer NR1.2: CTC AAG TGA GTC TGC CCC ATG CTG A and the reverse primer NR1.3as: CAC AGG GGA GGC AAC ACT GTG GAC F were used to amplify a 369 bp gene fragment for the *Grin1-2lox* allele and a 315 bp fragment for the wild type allele. Alternatively, the genotyping PCR of the EMMA mouse repository can be employed: https://www.infrafrontier.eu/sites/infrafrontier.eu/files/upload/public/pdf/genotype_protocols/EM09220_geno.pdf. The mice were bred and maintained group housed in the IBF Heidelberg. There were brought to the animal facility at the Central Institute of Mental Health Mannheim at the age of 10–14 weeks. To induce the Cre-mediated recombination at post-adolescence stages both *Grin1*^*f*/*f*^*/ErbB4-CreERT2* mice and control littermates were injected intraperitoneally twice a day with 100 μl (i.e., 1 mg) tamoxifen (T5648, Sigma-Aldrich) dissolved in 20 mg/ml peanut old, Sigma-Aldrich) for 5 days (36, 37) at the age of 7–8 weeks. After recovery the 10–14 days old mice were transferred to the behavioral facility (at the Central Institute of Mental Health in Mannheim) the mouse cohorts were subjected to the behavioral test battery for the next 2–3 weeks.

### Histological Analysis

Mice were anesthetized with isofluran (Baxter Healthcare Corporation) and perfused intracardiac with PBS and 4% paraformaldehyde (PFA, Merck) in PBS prior to decapitation (38). Brains were removed and fixed in ice-cold 4% PFA for 12 h, embedded in 2.5% agarose (Invitrogen) in PBS. After 12 h coronal vibratome sections (50 μm, Leica Vibratome VT100) were taken and transferred to a 24 ml well plate and in PBS. Slices were then briefly (1–5 min) counterstained in with DAPI (4′,6-diamidino-2-phenylindole, Thermo Fisher), 300 nM in PBS. Slices were washed 3–5 times with PBS. After final wash in PBS slices were mounted on glass slides (Menzel-Gläser), air dried for 10 min and embedded in aqua polymount (Polyscience). Overview images were acquired with an Axioimager/ Axiovision (Zeiss) and high-resolution images with the SP8 confocal microscope (Leica). Images were processed by Adobe Illustrator CS5 (Adobe).

### Behavioral Experiments

At the Central Institute of Mental Health Mannheim the animals were single-housed in Macrolon type II cages (26.8 × 21.5 × 14.1 cm) on a 12 h reversed dark-light cycle (lights on at 7 pm) and supplied with bedding (aspen wood ABEDD LTE E-002, ssniff-Spezialdiäten, Soest, Germany), nesting material (cotton square Zoonlab, Castrop-Rauxel) and water and food (LASQCdiet Rod16, Altromin, Lage) *ad libitum*. We assessed body weight once a week during cage changes under red light.

We assessed nesting behavior, locomotion and exploration (barrier test, open field and novel object test), anxiety (elevated o-maze, dark-light test), prepulse inhibition, cognition (radial arm maze, puzzle box, novel object recognition test) and stress coping (forced swim test). The behavioral observation started one week after the arrival with the observation of nesting in the home cage. Experiments were performed during the dark phase, at least 1 h after the light change, except for the nest test due to special demands. The mice were acclimatized to the testing room for at least 30 min, except for the FST, when acclimatization was limited to 6–10 min. Experimental equipment was cleaned after each trial with 70% ethanol. The testing order was of the mice was randomized for each behavioral test using randomizer.org. The experimenters were unaware of the genotype throughout the experimentation.

#### Nesting Test

Nest building was evaluated according to a rating scale on shape and cohesion of the nest as previously described (39). The mice were placed in a new home cage with cotton nestled 1 h before the onset of the dark phase and the score was determined 5 and 24 h later.

#### Barrier Test

The barrier test was performed as we described earlier (40). In brief, the mouse was introduced into the rear end of a clean Type III cage (42.5 x 27.6 x 15.3 cm) with reduced amount of bedding material. A transparent barrier (2 cm) separated the cage into two equal compartments. The setup was illuminated with 25 lux. The latency to cross the barrier, the number of crosses and the rearing were monitored.

#### Open Field and Novel Object Test

Locomotion was detected in a white open field (50 x 50 x 50 cm) illuminated by 25 lux, recorded by a video camera and analyzed by the imaging processing software Ethovision XT (Noldus Information Technology). Assessed parameters were total distance moved, center time (10 cm distance to the walls), movement and velocity (41). For the open field test the mouse was introduced to the center of the field for 10 min. In the subsequent novel object test, a water-filled 50 ml Falcon tube was introduced upside down in the center of the field. Latency and number of approaches to this novel object were counted manually for another 10 min.

#### Elevated o-Maze Test

To evaluate the approach-avoidance conflict in both mouse lines, the mice were introduced into the closed section of an o-shaped gray plastic runway (outer diameter 46 cm, width 6 cm, 50 cm of the ground). Two walled (height, 10 cm) sections of gray polyvinyl that were placed opposite to each other. The other sections were open. The floor was covered by grip tape to prevent falling. The latency to exit into the open arm, the time on the open arm and the number of crosses between the closed sections were monitored for 5 min.

#### Dark-Light Test

In another test for approach-avoidance conflict the mice were placed into the dark chamber (20 x 15 cm, black acryl with a black lid) of a 2-chamber box for 5 min. The latency to the first exit, the time spent in the light compartment and the number of exits into the chamber (30 x 15 cm, white acryl) illuminated with 600 lux was detected.

#### Acoustic Startle Response and Pre-pulse Inhibition

The mouse was introduced into a startle chamber (SR-LAB; San Diego Instruments) as previously described (37). Briefly, in the chamber a loudspeaker produced continuous background noise of 60 dB of sound pressure level (SPL) and the acoustic startle pulses (white noise, 115 dB SPL, 40 ms). After the acclimatization of 5 min, 5 initial startle stimuli were presented, followed by pseudorandomized presentation of pulse alone, control stimulus, pulse with prepulse (72 or 76 or 80 or 84 db, 100 ms before pulse) with 10 presentations of each trial type. The intertrial stimulus was randomized between 10 and 20 s. PPI was calculated as the percent decrease of the ASR magnitude in trials when the startle stimulus was preceded by a prepulse [100 x (mean ASR amplitude on pulse alone trials—mean ASR amplitude on prepulse-pulse trials)/mean ASR amplitude on pulse alone trials].

#### Radial Arm Maze

This learning task was performed as previously described (37). Briefly, the mouse was introduced into the center of a maze consisting of a central platform (20 cm in diameter) connected to eight arms (50 cm long, 8 cm wide), elevated 50 cm and covered with Plexiglas tunnels to permit visual orientation by extra-maze cues. The mouse was free to explore all arms and eat the bait (one millet seed) out of the food cups at the end of the arm for max. 10 min per day on 10 consecutive days. Otherwise, the session ended after the mouse ate all baits. Assessed movement parameters were distance moved, immobility, movement, time to complete and velocity, parameters on choices and errors were aborted trials, number of choices, correct choices, errors, procedural errors and working memory errors and angel choices. Working memory errors occurred when a mouse revisited an arm repetitively. The classification of working memory errors was based on the disparity to previous entries of the identical arm, ranging from 0 (re-entry) to max. 8 (more than eight entries in between were cumulated). Mice were tested for 10 day, with one run per day. The results of two consecutive days were given as one trial.

#### Puzzle Box Test

We assessed puzzle solving and memory as previously described in the puzzle box test (42). Briefly, the mouse was introduced into a brightly lit white chamber (58 × 28 cm, 600 lux) from where it could escape into a black goal zone (15 × 28 cm, covered with lid). The passage into the goal box was modified with increasing difficulty in the total trials on three consecutive days: run 1) open door over the underpass location; run 2–4, open underpath; run 5–7, underpath was filled with sawdust (bedding closed tunnel), and runs 8 and 9, underpath was blocked by a cardboard plug (blocked channel). A trial started by placing the mouse in the start zone and ended when all four paws of the mouse entered the goal zone or after a total time of 5 min. The performance of mice in the puzzle box was assessed by measuring the latency to enter the goal zone.

#### Novel Object Recognition

The novel object recognition was performed in the same setup as the open field test in a modified protocol (43). On a first day, the mouse was habituated to the arena for 10 min. On day two, the habituation of 10 min was repeated and followed by an exposure to two identical objects [either a transparent plastic cube (8 cm) standing on its tip filled with black paper in a frame made of coated clay or a glass candy jar filled with turquoise stones and a silver plastic lid (8 cm)] 2 h later for 7 min with at least 15 s of exploration to be included. Two hours later, the mouse was introduced again and was free to explore one familiar and one novel object for 5 min. Between the trials the mice were brought to their home cage. We assessed the time spent and the number of approaches exploring the objects.

#### Forced Swim Test

Mice were placed for 6 min into a glass cylinder (height 23 cm; diameter 13 cm) filled with water (21 °C) to a height of 12 cm. The latency to immobility and percentage of time spent immobile were determined by the image-processing system EthoVision XT, Noldus Information Technology (44, 45). This test was conducted twice, with a 24 h inter-trial interval.

#### Statistical Analyses

Statistical analyses were performed using SPSS Statistics version 24 (IBM, Armonk, NY). Differences were considered to be significant at a *P* < 0.05. The data were analyzed through two-way ANOVA with treatment and sex as factors or, when appropriate, by using repeated-measures ANOVA. Whenever no sex differences were observed, we merged the data of the groups (*n* = 14). No animals were excluded from the study. The sample size for all experiments was *n* = 7 per sex and genotype. The experimental unit was the single animal.

## Results

### Strategy for the ErbB4-CreERT2-Mediated GluN1 Expression

**Figure 1:**
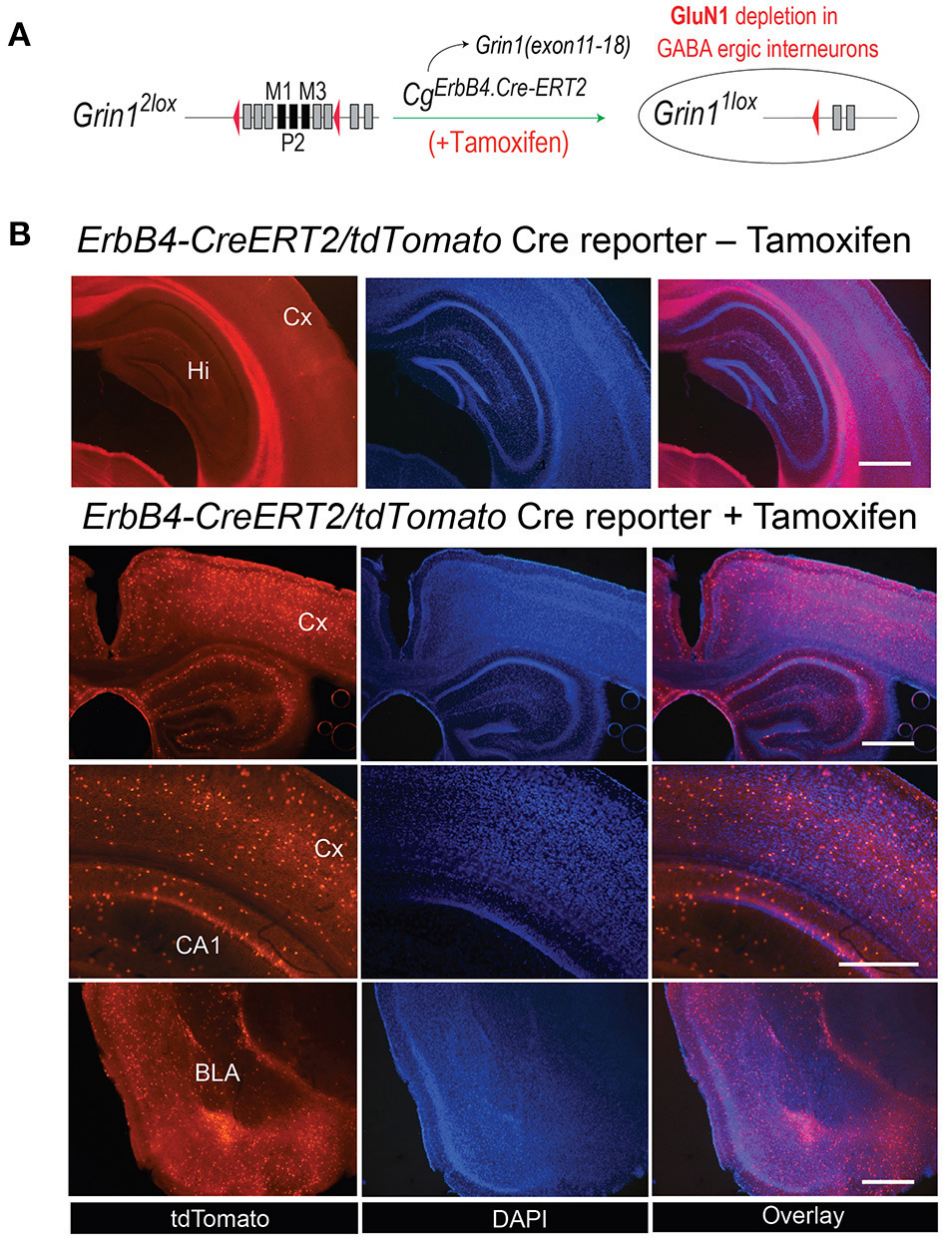
**(A)** Generation of *Grin1*^Δ*erbb*4^ mice and Cre-activirty in ErbB4 expressing cells. Schematic for the ErbB4-ERT2Cre-mediated deletion in *Grin1*^*f*/*f*^ mice. **(B)** The tamoxifen-induced expression pattern of the Cre-dependent tdTomato in B6. *Cg*^*Erbb*4*tm*1.1(*cre*/*ERT*2)*Aibs*/*J*^/*Gt*(*ROSA*) *26Sor*^*tm*14(*CAG*−*tdTomato*)*Hze*^ was evaluated in coronal brain sections of Tamoxifen-injected and in naive mice 3 weeks after Tamoxifen injection by the tdTomoto fuorescence in the DAPI stained section. Scale bars 1.0 mm, Hi, Hippocampus; Cx, Cortex; BLA, Basal lateral amygdala.

For our experimental approach of NMDAR deletion specifically in ErbB4 expressing neurons approach we selected the *Cg*^*ErbB*4*tm*1.1(*cre*/*ERT*2)*Aibs*/*J*^ for the tamoxifen-induced Cre expression (Figure 1A). In several previous studies this line was used reliably to study the *erbB4* gene expression in the mouse brain (46–48). Similarly, our gene-targeted floxed *Grin1* mice encoding the *Grin1*^*f*/*f*^ targeted allele was shown in our previous studies to be highly accessible for Cre-mediated inactivation (32) and for inducible inactivation later in development (33) or for PV knockout in PV-positive interneurons (49). For the demonstration of the cell type specific gene inactivation we employed the Cre-inducible tdTomato indicator mouse (Cg-*Gt(ROSA)26Sor*^*tm*14(*CAG*−*tdTomato*)*Hze*^) as this mouse line was used routinely to monitor the Cre activity in neuronal cell population e.g., (50). Thus coronal sections of our *Cg*^*ErbB*4*tm*1.1(*cre*/*ERT*2)*Aibs*/*J*^ Cg-*Gt(ROSA)26Sor*^*tm*14(*CAG*−*tdTomato*)*Hze*^ mice confirmed the Tamoxifen induced Cre expression in a subpopulation of neurons that was published before and that demonstrated the *erbB4* expression in a subpopulation of brain cells (Figure 1B, Supplementary Figure 1) which were previously described as interneurons and some glia cells (50) providing indirect evidence for Tamoxifen induced the deletions of NMDAR in those cells in our *Grin1*^*ff*^*/ErbB4-CreERT2* mice, similar to previous studies (23–25).

### Deletion of GluN1 in ErbB4-Expressing Cells During Adolescence Did Not Alter Basic Behavior

**Figure 2:**
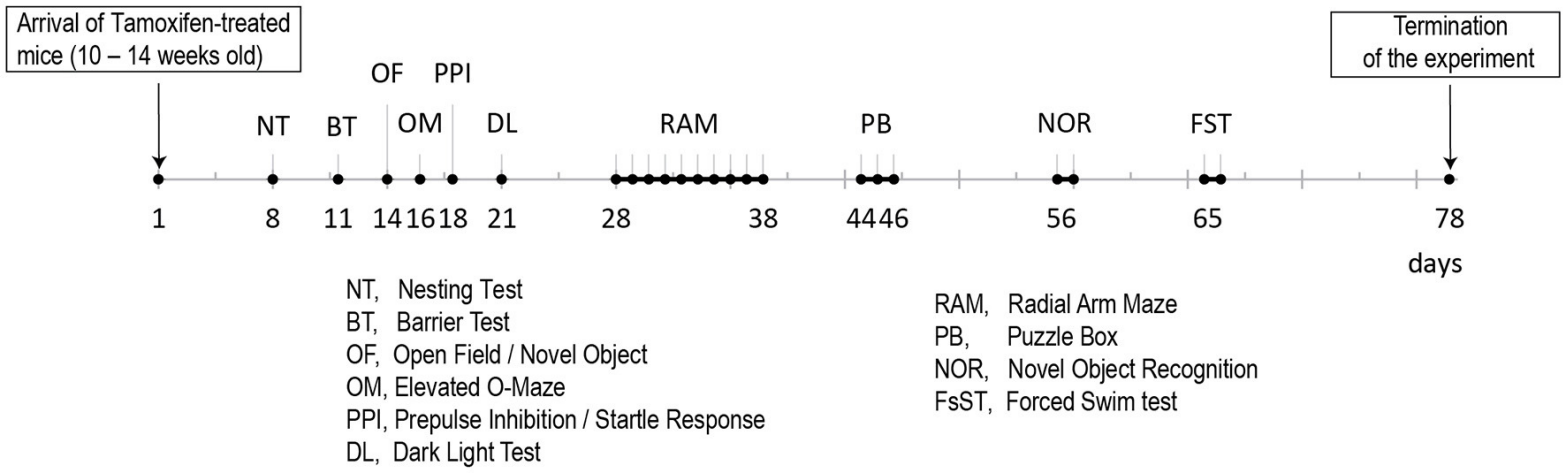
Time line for the behavioral analyses of the two Tamoxifen treated cohorts (B6. *Cg*^*Erbb*4*tm*1.1(*cre*/*ERT*2)*Aibs*/*J*^*/Gt(ROSA)26Sor*^*tm*14(*CAG*−*tdTomato*)*Hze*^ and control littermates).

**Figure 3:**
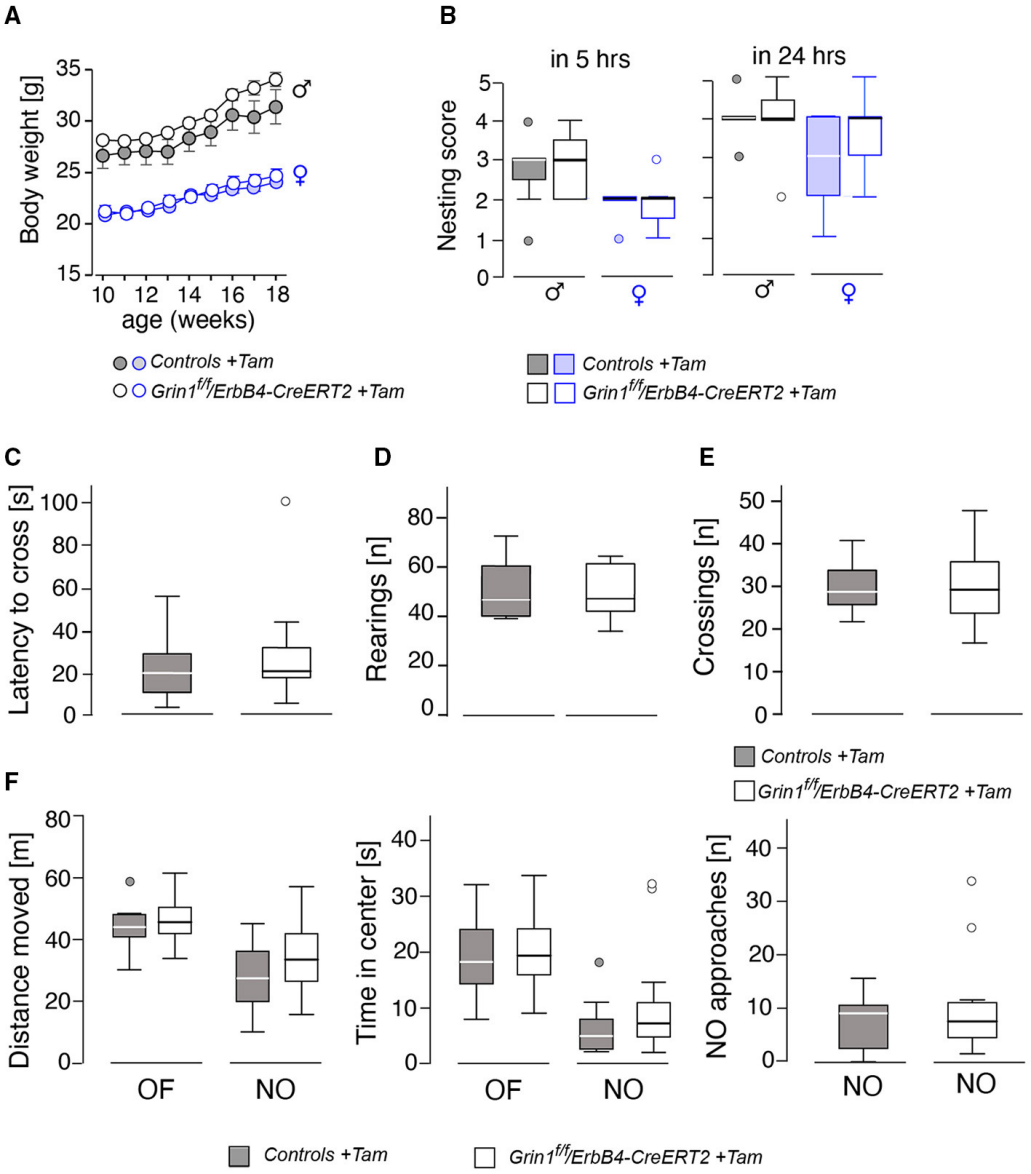
Assessment of basic physiological and locomotor parameters in male and female mice revealed no significant effects on the genotype. **(A)** Body weight, **(B)** nest building score after 5 and 24 h, **(C)** results of the barrier test in latency to cross the barrier, **(D)** number of rearings, **(E)** number of crossings over the barrier. In **(F)** the results of the open field (OF) and novel object (NO) test on gives the (left) the distance moved, (middle) time spent in center and (right) the approaches toward the novel object. Group size *n* = 14. Data is represented as means + SEM.

Behavioral testing of the animals was performed according to the time line given in Figure 2. We detected no differences in body weight (Figure 3A) due to genotype, but a time^*^genotype interaction F(8,192) = 2.384, *p* = 0.018, showing that *Grin1*^*ff*^*/ErbB4-CreERT2* increased faster in body weight than the controls. In addition, we found the typical body weight gain over time F(8,192) = 136.919, *p* < 0.001 and sex differences F(1,24) = 74.860, *p* < 0.001, as well as time^*^sex interactions F(8,192) = 9.895, *p* < 0.001 as the weight of the males increased quicker than the weight of the females. Nesting behavior also revealed a sex effect in the 5 h time window [5h: F(1,24) = 10.347, *p* = 0.004; 24 h: F(1,24) = 4.595, *p* = 0.042], but neither genotype effects nor interactions (Figure 3B). Locomotion and exploration were not affected by the genetic manipulation either, neither in the barrier test (genotype: number of rearings: F(1,27) = 0.062, *p* = 0.806; latency to cross: F(1,27)=0.247, *p* = 0.623; number of crosses F(1,27) = 0.098, *p* = 0.757) (Figures 3C–E) or the open field novel object test (genotype: open field (OF) distance moved: F(1,26) = 0.163, *p* = 0.690; novel object distance moved: F(1,26) = 2.502, *p* = 0.126; OF center time: F(1,26) = 0.394, *p* = 0.536; NO center time: F(1,26) = 2.394, *p* = 0.134; NO approaches: F(1,26) = 0.699, *p* = 0.411), (Figure 3F). Neither did we find sex specific differences or interactions in the Test (Figures 3C–F).

### Affective and Sensory-Gating Behavior Was Not Affected by the Genetic Manipulation

**Figure 4:**
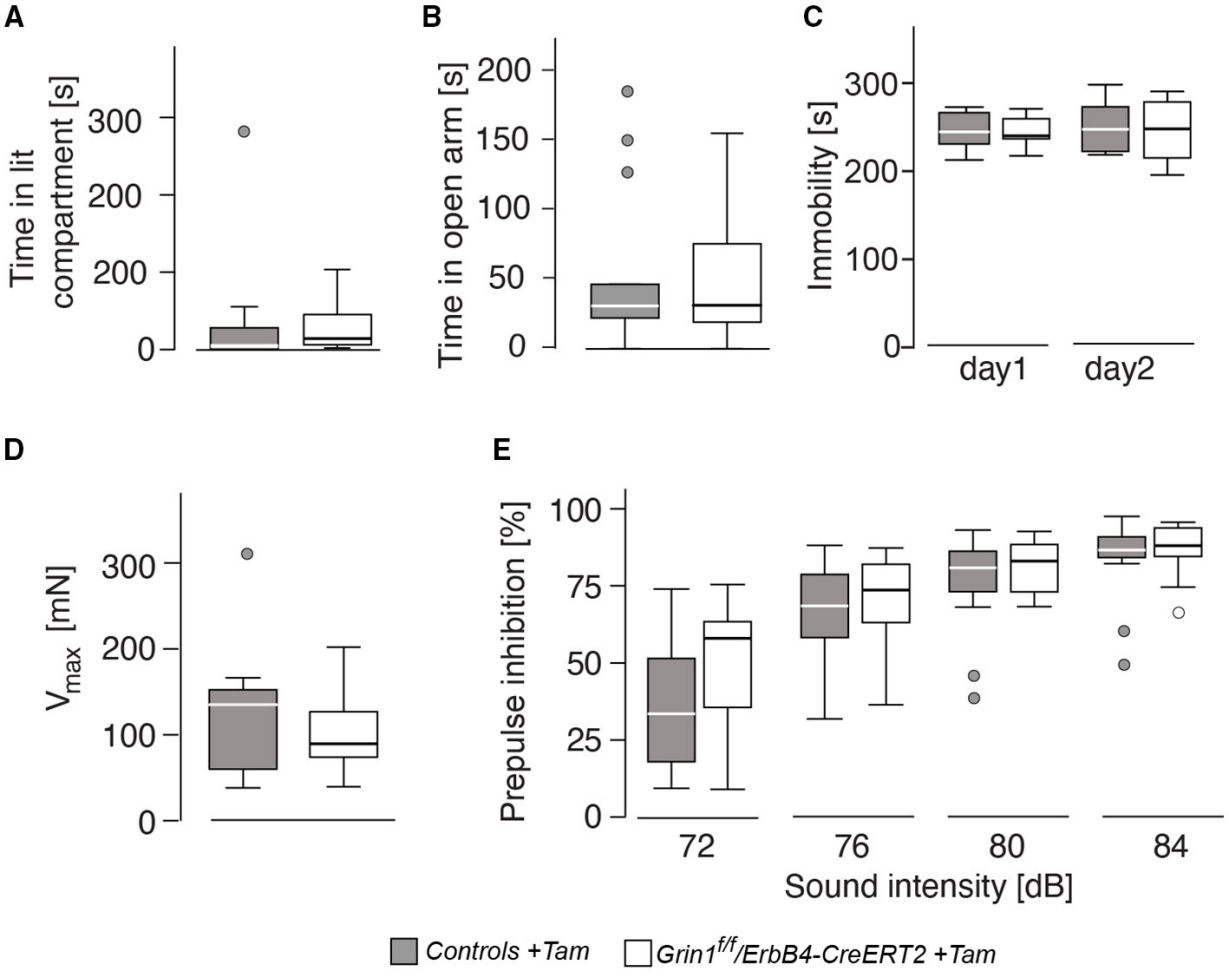
The genetic manipulation did not lead to alteration in affective behavior and prepulse-inhibition of the acoustic startle response. **(A)** Time spent in the illuminated part of the dark-light box, **(B)** time spent on the open arm of the elevated o-maze, **(C)** immobility in the forced swim test, **(D)** acoustic startle response and **(E)** prepulse inhibition of the startle response. Group size *n* = 7. Data is represented as means + SEM.

Anxiety-like behavior was similar in the dark-light test and the elevated o-maze (Figures 4A,B) for genotype and sex (genotype: time in lit compartment: F(1,26) = 0.141, *p* = 0.710; time on open arm: F(1,26) = 0.048, *p* = 0.829). Immobility, a coping behavior in the forced swim test, which is often associated with despair behavior and hence used as a marker for depressive-like behavior, was also not influenced by sex or genotype (genotype: immobility day 1: F(1,26) = 0.170, *p* = 0.684; immobility day 2: F(1,26) = 0.101, *p* = 0.753; Figure 4C). The acoustic startle response as well as the prepulse inhibition also displayed no differences between the factors (genotype: acoustic startle response: F(1,26) = 0.397, *p* = 0.534; intensity: F(3,78) = 100.971, *p* < 0.001; genotype: F(1,261 = 0.669, *p* = 0.208); Figures 4D,E). We found an intensity^*^genotype interaction F(3,78) = 2.689, *p* = 0.052, which indicates a tendency to lower responsivity to the different noise intensities in *Grin1*^*ff*^*/ErbB4-CreERT2* mice.

### Learning and Memory Was Not Affected by the ErbB4-CreERT2-Induced NMDAR Knockout

**Figure 5:**
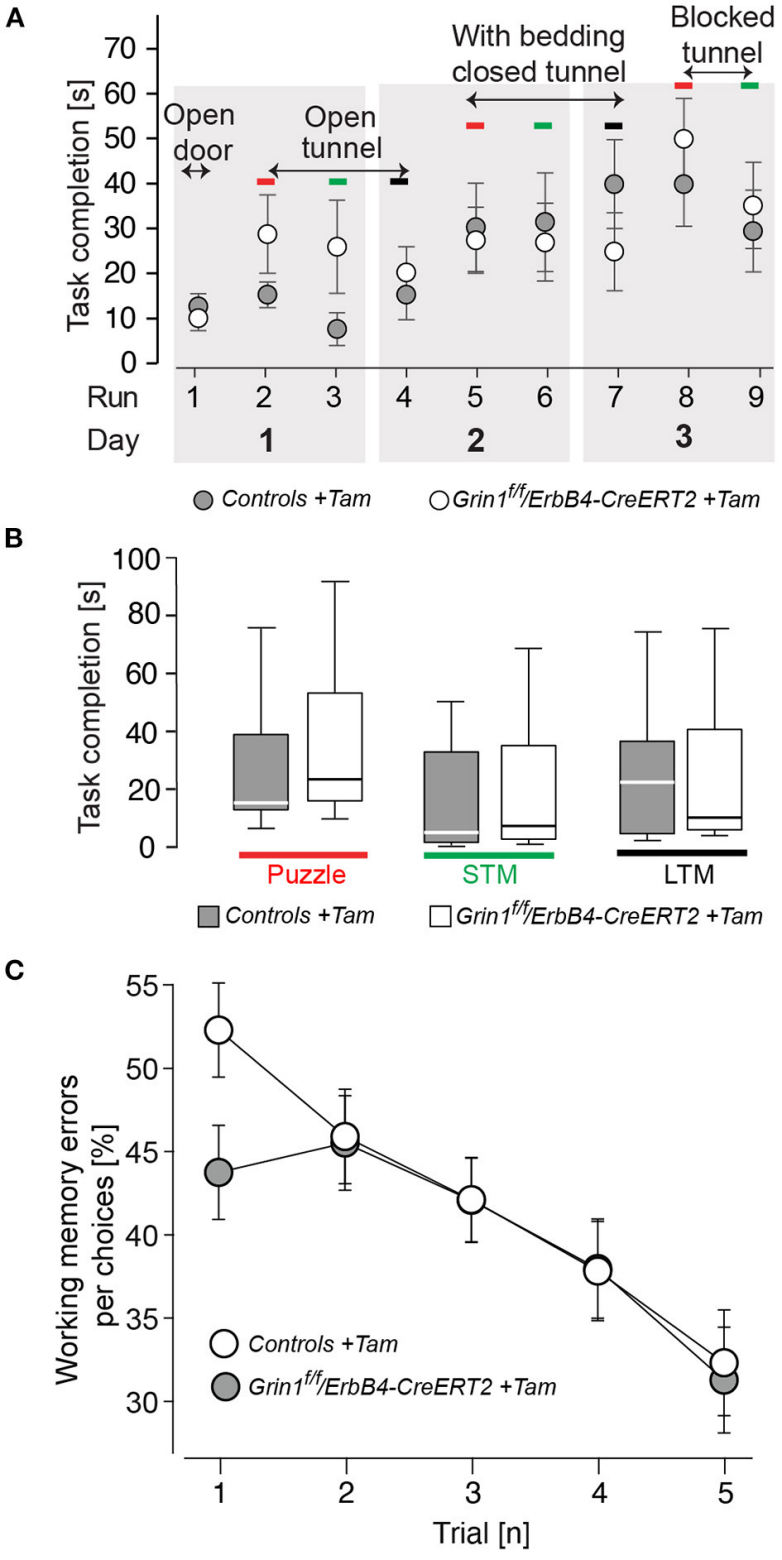
Mutant mice showed no impairments in learning and puzzle solving. **(A)** Time to complete the puzzle per trial due to condition. **(B)** Puzzle, analysis of the recognition of a novel task; STM, short-term memory (repetitive task on the same day); LTM, long-term memory (repetitive task of the last day). **(C)** Working memory errors in the radial maze in 5 consecutive trials (1 tial = 2 runs). Controls are shown in black, *Grin1*^Δ*ErbB*4*f*/*f*//*ErbB*4−*CreERT*2^ in white. Group size *n* = 14. Data is represented as means + SEM.

Since in the novel object recognition was normal *Grin1*^*ff*^*/ErbB4-CreERT2* mice (Supplementary Table S1) we analyzed the learning behavior in our mutant mice in more detail, in order to detect shuttle differences in complex attentional tasks: the puzzle box and in the radial maze (Figure 5). In our analysis we found that in learning in all tasks of the puzzle box of *ErbB4-CreERT2-*mice was comparable to control littermates (Figure 5A). Moreover, when the conflict solution (puzzle), the short term (STM) or long term (LTM memory was analyzed we could not find a statistical difference between genotypes [genotype: puzzle: F(1,26) = 0.540, *p* = 0.469; STM: F(1,26) = 0.675, *p* = 0.390; LTM: F(1,26) = 0.241, *p* = 0.628; Figure 5B]. Similarly, in the spatial radial maze (RAM) we detected no increased working memory errors in *Grin1*^*ff*^*/ErbB4-CreERT2* mice compared to controls during the acquisition of the task [genotype: F(1,26) = 0.517, *p* = 0.478; Figure 5C] indicating that the *Grin1*^*ff*^*/ErbB4-CreERT2* are not impaired in responses to natural stimuli.

## Discussion

Here we report that ablation of GluN1-containing NMDAR in ErbB4 expressing cells in adults mice does not significantly affect cognition and does not induce the typical behavioral correlates of schizophrenia, depression and anxiety. To our knowledge, our study provides the first characterization of a genetic model of inducible genetic ablation of NMDAR during late adolescence in neurons expressing the NRG1 receptor ErbB4, with relevance for psychiatric disorders, considering that NRG1 and ErbB4 are main candidate risk genes gene for schizophrenia (26).

The present results appear at a first glance surprising since mutant mice heterozygous for either NRG1 or ErbB4 show a behavioral phenotype that resembles alterations seen in schizophrenia and, furthermore, NRG1 hypomorphs, expressing 50% of the normal levels of NRG1, have 16% fewer functional NMDARs than wild-type mice (26). However, as mentioned by these authors, such results have to be interpreted with caution so that they do not necessarily mean that the principal pathogenic alteration in schizophrenia lies in the glutamate system (26). One important aspect that needs to be taken into consideration refers to the fact that NMDAR expression is affected already in early brain development in the NRG1 hypomorph mice, whereas they are ablated only postnatally in our inducible pharmacogenetic model. As mentioned previously, only early postnatal, but not early adult ablation of NMDAR in (mainly, but not exclusive) PV-positive interneurons triggers psychosis-like changes (25), causing an excitation-inhibition E/I imbalance which emerges after adolescence concomitantly with significant dendritic retraction and dendritic spine re-localization in pyramidal neurons (51). One possible explanation could be that NMDA currents gradually decrease and even became undetectable during cortical development, with most (74%) of the parvalbumin-positive interneurons exhibiting no NMDA current in adults, in contrast to other interneuronal populations, where they remain stable (52). Therefore, an early postnatal ablation of NMDARs appears crucial in inducing protracted neuroplastic impairment that underlies schizophrenia-associated abnormalities. We cannot exclude that ablation of NMDAR in ErbB4-positive cells induced at earlier time-points than in the present investigation may trigger schizophrenia-like abnormalities. Future studies should determine and compare such stage-dependent effects of cell type-restricted NMDAR genetic manipulation.

Our data indicate that post-adolescent deletion of NMDAR even extended to a much larger neuronal population than PV-positive interneurons is insufficient to trigger behavioral changes associated with psychosis. The identification of the neural substrate of these alterations is not yet finalized, other brain regions such as thalamic neurons (53) or other interneuronal subpopulations, such as those expressing somatostatin (54), are as well valid candidates. Another possibility is that NMDAR deficiency in PV and possibly ErbB4 neurons may be a risk factor for developing schizophrenia, but is not sufficient on its own: environmental risk factors or other supplementary triggers may be needed to lead to clinical manifestation (50). In line with this view is as well the finding that global pharmacological blockade of NMDAR with MK-801 induces catatonia-like changes, as a feature both of a severe schizophrenia and anti-NMDAR encephalitis, in *Grin1*^Δ*PV*^ mice (34).

### Limitations of the Study

Finally, we wish to mention that the validation of the current inducible pharmacogenetic model is limited by various factors. Providing experimental evidence for the quantitative removal of NMDAR from cells expressing the *erbB4* gene in animal models with cell type specific deletions using the *erbB4-CreERT2* knockin line is a big experimental challenge. In previous mice with interneuron-restricted NMDAR depletion (*Grin1* cKOs), the authors used single cell electrophysiology to demonstrate the loss of NMDAR currents, which complemented the demonstration that the CRE expression was restricted to interneurons using Cre-indicator mice (23). In our conditional NMDAR knock out mouse model inducible deletion was started not early postnatally as in that model, but at post-adolescent stages, requiring functional analysis at later, adult time points. However, preparing consistently healthy acute brain slices from mature animals for patch clamping experiments is challenging, due to extensive myelination, reduced tissue viability and increased vulnerability to damage etc., the vast majority of brain electrophysiologists working with brain slices from juvenile animals. Therefore, a reliable electrophysiological single call analysis is very difficult to be performed due to the technical limitations of single cell patch analysis of adult mice. Hence we relied–as all of previous studies, on the Cre-dependent tdTomato expression pattern induced in our mice, which was used before for efficient Cre-dependent removal of the NMDAR. For our studies, we have specifically imported the Erbb4tm1.1(cre/ERT2)Aibs/J mouse line from Jackson Labs to Heidelberg and used it in our experiments. We selected this line because it has already been successfully used in multiple studies. Thus, the functional tamoxifen-induced Cre recombinase activity in Rosa Cre-Indicator A14 mice was reproducible and also clearly detected in erbB4-positive cells (35, 50, 55). In addition, we used our floxed GluN1 mice, which we used successfully in three manuscripts (32–34), indicating that the Cre-mediated inactivation of our floxed *Grin1*^*f*^ allele is efficient.

In this context, it is important to mention that the detection of successful conditional Cre-induced gene ablation of highly expressed CNS specific genes, such as *Grin1* in a small population of widely scattered cells in the CNS, such as here the ErbB4-positive neurons, is experimentally challenging. Belforte et al. has succeeded in using double *in situ* hybridization to detect the loss of NMDAR in most GAD67-positive interneurons in S1 somatosensory cortex (25), although NMDARs are tightly distributed in the CNS (32, 56). For the electrophysiological NMDAR analysis in the GAD67-positive cells he adopted a method that was initially developed to determine the expression profile in single 5HT3A1 expressing cells in the mouse brain. In this method, the 5HT3A neurons were tagged by the a fluorescent protein (FP). By Laser Capture Microscopy (LCM) the RNA of the FP positive cells was isolated and the mRNA was amplified by single cell RT-PCR. In this example, the gene expression profiles of EGFP-tagged 5HT3A expressing neurons was determined (57). To date several publicly available “Fluorescent Cre-activity indicator mouse lines” (see Jackson labs, and the A14 line used in this study) are available. Their usage have greatly facilitated the specialized task of detecting Cre expressing single cells in brain slices. By using one of those CRE-FP transgenes Belforte et al., was able to detect the loss of NMDAR currents in CRE-FP expressing GAD67 interneurons of young mice (25) and Lin et al., succeeded in determining the electrophysiological profile of vGat deficient ErbB4 cells (58). Thus, the implementation of combined CRE-FP in in the same cell opened the possibility of optimal, reliable electrophysiological analysis of gene defects in sparse neuronal subpopulations. A lot of patience and breeding effort is required here to cross three different mouse lines. However, this cellular electrophysiological analysis appears to be largely limited to brain slices from young mice. Thus, Belforte et al. also show E-phys patching of Cre-FP-expressing GAD67 cells only in young mice but not in old mice from an independent cohort of a second NMDAR-KO mouse line (25). For adult mice LCM the RNA of single cells is still an option.

In conclusion, our results showing that restricted post-adolescent deletion of NMDAR from a relatively large neuronal population of ErbB4-positive neurons does not affect behavior is once again emphasizing the role of neurodevelopmental impairment in the emergence of several psychiatric disorders. Inducible genetic models represent useful tools toward identifying the neuronal populations implicated in NMDAR-driven psychosis at specific developmental stages, including adulthood.

## Data Availability Statement

The original contributions presented in the study are included in the article/Supplementary Material, further inquiries can be directed to the corresponding author.

## Ethics Statement

All experimental procedures were approved by the Animal Welfare Committee (Regierungspräsidium Karlsruhe) and carried out according to the European Communities Council Directive 63/2010/EU (license number: 35-9185-81-G-3-17).

## Author Contributions

AM, PG, and DI designed the study, analyzed the results, and wrote the manuscript. MV, SC, and RS generated, bred, and analyzed the transgenic animal lines. NP and AM performed the behavioral analyses. All authors contributed to the article and approved the submitted version.

## Funding

The present work was supported by grants from the Deutsche Forschungsgemeinschaft (DFG) IN 168/3-1, the Ingeborg Ständer Foundation, the ERA-NET NEURON program, the Bundesministerium für Bildung und Forschung (BMBF) under the frame of Neuron Cofund (ERA-NET NEURON NMDAR-PSY) and the Swiss National Foundation (SNF) 186346 to DI.

## Conflict of Interest

The authors declare that the research was conducted in the absence of any commercial or financial relationships that could be construed as a potential conflict of interest.

## Publisher's Note

All claims expressed in this article are solely those of the authors and do not necessarily represent those of their affiliated organizations, or those of the publisher, the editors and the reviewers. Any product that may be evaluated in this article, or claim that may be made by its manufacturer, is not guaranteed or endorsed by the publisher.
