# Supplementary material for: Cre-Activation in ErbB4-Positive Neurons of Floxed Grin1/NMDA Receptor Mice Is Not Associated With Major Behavioral Impairment
Source: Front Psychiatry. 2021 Nov 25;12:750106. doi: 10.3389/fpsyt.2021.750106 (PMC8660629; doi:10.3389/fpsyt.2021.750106)
Supplement: Supplementary file 1 [file Table_1.DOCX]

|  | Sex | genotype | Sex*genotype |
| --- | --- | --- | --- |
| Side preference | n.s. | n.s. | n.s. |
| Novelty preference - approaches | n.s. | n.s. | F(1,27)=3.297, p=0.082 |
| Novelty preference - time | F(1,27)=3.181 p=0.081 | n.s. | n.s. |

Supplementary table 1: No significant differences were found in side preference (=time spent on left or right object/ total time during the phase with two identical objects), preferences to approach the novel object (=number of preferences towards the novel object/total approaches) and the preference to time spent to investigate the novel object (=time spent on novel object/total time).
