# Supplementary material for: Cre-Activation in ErbB4-Positive Neurons of Floxed Grin1/NMDA Receptor Mice Is Not Associated With Major Behavioral Impairment
Source: Front Psychiatry. 2021 Nov 25;12:750106. doi: 10.3389/fpsyt.2021.750106 (PMC8660629; doi:10.3389/fpsyt.2021.750106)
Supplement: Supplementary file 2 [file Table_2.DOCX]

Supplementary figure legend

Supplementary Figure 1. The tamoxifen-induced expression pattern of the Cre-dependent tdTomato in *Cg^Erbb4tm1.1(cre/ERT2)Aibs/J^/ Gt(ROSA)26Sor^tm14(CAG-tdTomato)Hze^* was evaluated 2 weeks after Tamoxifen injection in coronal brain sections of Tamoxifen-injected and in naive mice. Vibratome sections (80 mm) were stained with DAPI and imaged in the blue and red channel by the Axiomager 1 (Zeiss).
