# Supplementary figures and images for: Cre-Activation in ErbB4-Positive Neurons of Floxed Grin1/NMDA Receptor Mice Is Not Associated With Major Behavioral Impairment
Source: Front Psychiatry. 2021 Nov 25;12:750106. doi: 10.3389/fpsyt.2021.750106 (PMC8660629; doi:10.3389/fpsyt.2021.750106)

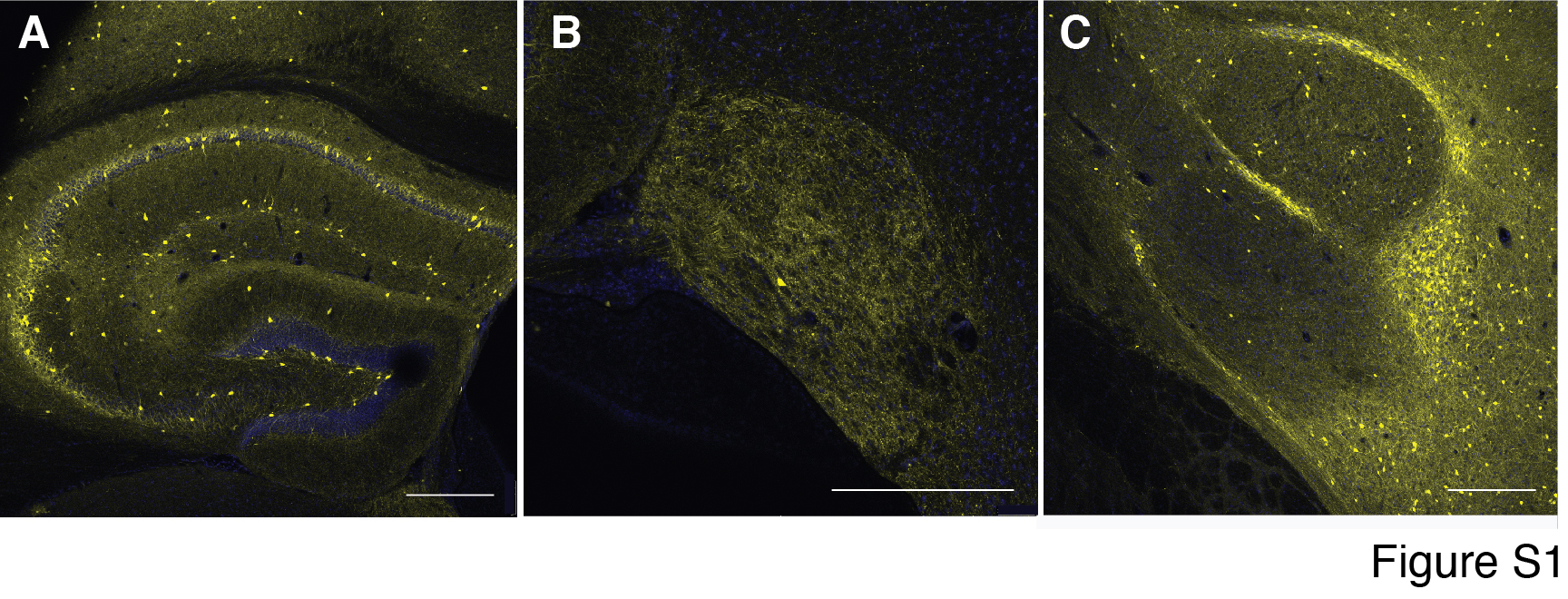

Supplement: Supplementary file 3 [file Image_1.JPEG]
